# Optimized dual-energy CT protocol for inferior vena cava CT angiography using 60 keV virtual monoenergetic imaging and 60% ASIR-V

**DOI:** 10.3389/fcvm.2026.1969018

**Published:** 2026-10-29

**Authors:** Jiantao Deng, Xiaoping Fan, Qingwei Zhang, Jing Yan, Yong Li, Gaowu Yan, Linwei Zhao, Xiaocheng Shen, Qijia Peng, Ting Ma, Siyi Wu, Xiaohan Wang, Xisi HE, Morgan A. McClure, Anup Bhetuwal

**Affiliations:** 1 Department of Radiology, Suining Central Hospital, Suining, China; 2 Department of Radiology, Affiliated Hospital of North Sichuan Medical College, Nanchong, China; 3 Department of Radiology, Affiliated Hospital of Southwest Medical University, Luzhou, China; 4 Department of Radiology and Imaging, Institute of Rehabilitation and Development of Brain Function, The Second Clinical Medical College of North Sichuan Medical College Nanchong Central Hospital, Nanchong, China; 5 Sichuan Key Laboratory of Medical Imaging and Department of Radiology, Affiliated Hospital of North Sichuan Medical College, Nanchong, China

**Keywords:** adaptive statistical iterative reconstruction-V, computed tomography, CT angiography, dual-energy CT, inferior vena cava, iodinated contrast medium, radiation dose, virtual monoenergetic imaging

## Abstract

**Objective:**

To investigate the effects of a dual-energy CT (DECT) protocol combining virtual monoenergetic imaging (VMI) with adaptive statistical iterative reconstruction-V (ASIR-V) on image quality and radiation dose for inferior vena cava (IVC) CT angiography (CTA), and to compare the optimized protocol with a conventional 120 kVp protocol.

**Methods:**

120 consecutive patients undergoing IVC-CTA were enrolled and allocated sequentially to two groups (*n* = 60 each). Group A underwent conventional 120 kVp scanning with 400 mgI/mL contrast medium at 1.5 mL/kg and 50% ASIR-V reconstruction. Group B underwent DECT (rapid 80/140 kVp switching) with 320 mgI/mL contrast medium at 1.2 mL/kg. In Group B, seven VMI datasets from 40 to 70 keV in 5 keV increments were first reconstructed with 50% ASIR-V. At 60 keV, images were reconstructed with filtered back projection (FBP; 0% ASIR-V) and ASIR-V levels from 20% to 100% in 20% increments. Objective image quality was assessed using CT attenuation, standard deviation (SD), background noise, signal-to-noise ratio (SNR), and contrast-to-noise ratio (CNR) at five venous levels. Two radiologists independently rated subjective image quality and diagnostic confidence on 5-point Likert scales. Dose-length product (DLP), effective dose (ED), contrast medium volume, and iodine intake were also recorded and compared between protocols.

**Results:**

In Group B, with ASIR-V fixed at 50%, CT attenuation, SD, background noise, SNR, and CNR decreased progressively as VMI energy increased. Among the seven VMI levels, 60 keV achieved the highest subjective image-quality score (all *P* < 0.05). At 60 keV, increasing ASIR-V reduced vascular SD and background noise and increased SNR and CNR. The 60 keV VMI plus 60% ASIR-V combination (Group B1) achieved the highest subjective score. Compared with Group A, Group B1 had significantly higher IVC CT attenuation and background noise (both *P* < 0.001), but SNR, CNR, and subjective image-quality scores did not differ significantly (all *P* > 0.05). Diagnostic-confidence scores for thrombosis, luminal stenosis, post-filter placement, and normal IVC findings were similar between Groups A and B1 (all *P* > 0.05). Relative to Group A, Group B1 reduced contrast medium volume, iodine intake, and ED by approximately 20.0%, 36.0%, and 12.51%, respectively (all *P* < 0.001).

**Conclusion:**

The DECT protocol combining 60 keV VMI with 60% ASIR-V provided a favorable balance of image quality for IVC-CTA. The optimized protocol achieved SNR, CNR, subjective image quality, and diagnostic confidence similar to those of the conventional 120 kVp protocol while reducing contrast medium volume, iodine intake, and effective radiation dose.

## 1 Introduction

The inferior vena cava (IVC) returns approximately two-thirds of the venous blood from the abdominopelvic organs and lower extremities to the right atrium. Congenital variants (e.g., absence of the hepatic segment, duplicated IVC, and left-sided IVC) and acquired abnormalities (e.g., tumor invasion, Budd-Chiari syndrome, and thrombosis) may occur in the IVC (1, 2). Accurate recognition of these variants and abnormalities is therefore important for diagnosis, treatment planning, and follow-up. Owing to its noninvasive nature, high spatial resolution, and rapid acquisition, CT angiography (CTA) has become a preferred noninvasive imaging modality for evaluating IVC disease (3, 4).

Conventionally, 120 kVp protocols combined with high-concentration iodinated contrast media (usually 350–400 mgI/mL) are widely used and provide relatively stable image quality. However, clear depiction of the IVC lumen and lesion margins often requires high-concentration contrast media administered at relatively large volumes, potentially increasing the risk of contrast-induced acute kidney injury (CI-AKI), particularly in patients with impaired renal function or those requiring repeated examinations (5, 6). Lowering the tube voltage can improve vascular enhancement but may increase image noise and reduce diagnostic confidence (7, 8). In addition, conventional polychromatic images may provide limited contrast for delineating IVC lesions such as intraluminal thrombus or tumor invasion. Thus, reducing contrast medium volume, iodine load, and radiation dose while maintaining diagnostic image quality has become an important goal in optimizing IVC-CTA protocols.

Dual-energy CT (DECT) provides additional opportunities for vascular imaging beyond conventional polychromatic CT. For example, on a Revolution CT system, rapid kVp-switching technology can generate 101 virtual monoenergetic image (VMI) datasets from 40 to 140 keV. Low-energy images (40–70 keV) substantially increase iodine attenuation and vascular contrast (9, 10), whereas higher-energy images (80–140 keV) can reduce beam-hardening artifacts and image noise (11). Adaptive statistical iterative reconstruction-V (ASIR-V) can further reduce image noise and improve signal-to-noise ratio (SNR) (12, 13). Previous studies have shown that combining VMI with iterative reconstruction in vascular CTA may reduce both contrast medium requirements and radiation exposure while preserving image quality (14–17). However, most studies of IVC-CTA have focused on optimizing a single parameter, such as the VMI energy level or reconstruction algorithm, or have simply combined two techniques (4, 18). A systematic strategy integrating reduced contrast concentration and volume, optimal VMI energy, and optimal ASIR-V strength, followed by comprehensive comparison with a conventional 120 kVp protocol, remains insufficiently studied.

Accordingly, this study aimed to evaluate the feasibility of an optimized IVC-CTA protocol that integrates DECT, a reduced-concentration/reduced-volume contrast strategy, VMI, and ASIR-V. We assessed how different VMI energy levels and ASIR-V strengths affect objective and subjective image quality, identified the most favorable VMI and ASIR-V combination, and compared the resulting optimized protocol with a conventional 120 kVp protocol in terms of image quality, iodine intake, and radiation dose.

## 2 Materials and methods

### 2.1 Study population

This was a single-center, prospective, non-randomized cohort study with sequential allocation by study period. The study was approved by the Ethics Committee of Suining Central Hospital (approval no. KYLLKS20250094), and written informed consent was obtained from all patients or their legal guardians.

Between January and December 2025, 150 consecutive patients scheduled for IVC-CTA were prospectively screened. The inclusion criteria were: (a) age ≥18 years; (b) clinically suspected IVC disease or the need for follow-up; and (c) voluntary participation in this study. The exclusion criteria were: (a) diffuse stenosis involving the IVC (*n* = 5); (b) severe IVC occlusion (*n* = 7); (c) disease causing substantial disturbance of the venous circulation (*n* = 8); (d) severe motion artifacts that precluded diagnostic evaluation (*n* = 6); or (e) non-diagnostic CTA image quality (*n* = 4). Of the 150 patients screened, 30 were excluded, and 120 were included in the final analysis.

The sample size was calculated based on the primary outcome measure of SNR of the IVC. Assuming a mean difference of 12 and a standard deviation of 20 based on previous studies, a two-sided two-sample *t*-test with *α* = 0.05% and 80% power required at least 45 patients per group. To account for potential dropouts and non-diagnostic images, we planned to enroll 60 patients per group. The patient selection process is shown in Supplementary Figure S1.

Patients were allocated according to the chronological implementation of the clinical scanning protocols. Patients enrolled from January through June 2025 underwent the conventional protocol (Group A), whereas those enrolled from July through December 2025 underwent the DECT protocol (Group B), with 60 patients in each group. Group A included 33 men and 27 women (mean age, 63.88 ± 9.42 years; range, 49–80 years), and Group B included 35 men and 25 women (mean age, 64.31 ± 10.33 years; range, 45–81 years). No significant between-group differences were observed in age, sex, height, weight, body mass index (BMI), or IVC disease category (all *P* > 0.05; Table 1).

**Table 1:** Demographic and clinical characteristics of the study population.

Patient characteristic	Group A (*n* = 60)	Group B (*n* = 60)	*P* value
Age (years)	63.88 ± 9.42	64.31 ± 10.33	0.811
Weight (kg)	63.19 ± 4.80	63.48 ± 6.66	0.784
Height (cm)	163.85 ± 2.86	163.93 ± 4.50	0.904
BMI (kg/m^2^)	23.52 ± 1.44	23.62 ± 2.31	0.774
Sex (male/female)	33/27	35/25	0.854
IVC status, *n* (%)			
Thrombosis	12 (20.0)	15 (25.0)	0.512
Stenosis	18 (30.0)	15 (25.0)	0.540
IVC filter placement	21 (35.0)	18 (30.0)	0.559
Normal	9 (15.0)	12 (20.0)	0.471

IVC, inferior vena cava.

### 2.2 Data acquisition

All examinations were performed on a 256-row DECT scanner (Revolution CT; GE Healthcare, Waukesha, WI, USA). Patients were positioned supine, feet first, with both arms extended above the head. Scanning was performed craniocaudally from the diaphragmatic dome to the inferior margin of the pubic symphysis to ensure complete coverage of the IVC and its major tributaries, including the renal, common iliac, and femoral veins. Group A (conventional protocol) underwent helical scanning at 120 kVp using Iomeprol at 400 mgI/mL (Bracco Sine Pharmaceutical Co., Ltd., Shanghai, China) at 1.5 mL/kg. Group B (DECT protocol) underwent gemstone spectral imaging (GSI) with rapid 80/140 kVp switching using iodixanol at 320 mgI/mL (Yangtze River Pharmaceutical Group Co., Ltd., Jiangsu, China) at 1.2 mL/kg. Other acquisition parameters were identical between groups: rotation time, 0.5 s; pitch, 0.992:1; detector coverage, 80 mm; display field of view, 320 mm; matrix, 512 × 512; automatic tube-current modulation (Smart mA for the conventional protocol and GSI Assist for the spectral protocol); noise index, 10; slice thickness, 5 mm; reconstruction interval, 5 mm; standard reconstruction kernel; and 50% pre-reconstruction ASIR-V. Iodinated contrast medium was administered through an antecubital vein using a dual-head power injector (Insight C12, Nanjing Jusha Display Technology Co., Ltd., Jiangsu, China) at 4.0 mL/s, followed by a 30 mL saline flush at the same rate. Smart Prep bolus tracking was used, with a region of interest (ROI) placed in the main portal vein at the hepatic hilum. Monitoring began 15 s after contrast injection; when attenuation reached 100 HU, IVC-CTA was automatically initiated after an additional 45 s delay. The scanner-reported dose-length product (DLP, mGy·cm) was recorded for each patient. Effective dose (ED, mSv) was calculated as ED = DLP × k, where *k* = 0.015 mSv/(mGy·cm), the abdominal conversion coefficient recommended in the European guidelines on quality criteria for computed tomography. Contrast medium volume was recorded, and iodine intake was calculated as iodine intake (g) = iodine concentration (mgI/mL) × contrast medium volume (mL) × 0.001.

### 2.3 Image reconstruction

Axial images were reconstructed from the raw data using a window width of 350 HU and a window level of 40 HU, with slice thickness and reconstruction interval both set to 1.25 mm. In Group A, images were reconstructed with 50% post-reconstruction ASIR-V. In Group B, a 120 kVp-like polychromatic dataset was generated automatically. Seven VMI datasets from 40 to 70 keV in 5 keV increments were then reconstructed with 50% post-reconstruction ASIR-V to identify the optimal monoenergetic level. At the selected energy level, images were first reconstructed with filtered back projection (FBP; 0% ASIR-V) and then reconstructed with ASIR-V at 20%, 40%, 60%, 80%, and 100% to determine the optimal reconstruction strength. All datasets were transferred to the picture archiving and communication system (PACS) and an AW 4.7 workstation (GE Healthcare, USA) for further analysis. A chief radiologic technologist performed volume rendering (VR), multiplanar reconstruction (MPR), and maximum intensity projection (MIP) from the source axial images on the workstation.

### 2.4 Objective image analysis

Objective image analysis was performed on an AW 4.7 workstation by two radiologists with 12 and 15 years of CTA interpretation experience, respectively. The ROIs for objective image measurements were determined by consensus between the two radiologists. After agreement on ROI placement was reached, the measurements were performed on the consensus ROIs, and a single set of objective measurements was used for analysis. Therefore, inter-reader agreement was not calculated for the objective measurements. (1) On thin-section axial images, circular ROI 1 was manually placed within the hepatic, suprarenal, and infrarenal segments of the IVC and within the common iliac and femoral veins to measure venous CT attenuation and standard deviation (SD). Each ROI covered more than two-thirds of the cross-sectional luminal area whenever feasible. (2) At the same axial level, circular ROI 2 was placed in adjacent muscle (erector spinae or iliopsoas) to measure muscle attenuation and SD; muscle SD was used as background noise. (3) Measurements were obtained on three consecutive slices, and the mean of the three measurements was used for analysis. ROIs were positioned to avoid thrombus, filter-related artifacts, calcification, and vessel margins. For images reconstructed with the same algorithm at different energy levels, and for images reconstructed with different algorithms at the same energy level, ROI position, size, and shape were kept identical. The IVC signal-to-noise ratio (SNR) and contrast-to-noise ratio (CNR) were calculated as follows: SNR = CT_vessel/SD_vessel; CNR = (CT_vessel—CT_muscle)/SD_muscle. The ROI placement method is illustrated in Supplementary Figure S2.

### 2.5 Subjective image quality assessment

The same two radiologists independently evaluated axial and post-processed images using a 5-point Likert scale while blinded to the reconstruction and acquisition settings. Before review, all images were anonymized and stripped of patient identifiers, clinical information, and scan parameters, including keV level and ASIR-V strength. Images reconstructed at different keV levels and ASIR-V strengths were randomly ordered and presented individually; the readers were unaware of image labels, reconstruction parameters, patient information, and quantitative measurement results. Image quality was graded as follows: 5 = excellent, with very homogeneous vascular enhancement, very clear anatomic structures, no appreciable noise, and full diagnostic acceptability; 4 = good, with homogeneous enhancement, clear anatomy, low image noise, and diagnostic quality; 3 = acceptable, with generally homogeneous enhancement, adequately depicted anatomy, moderate image noise, and sufficient quality for basic diagnosis; 2 = poor, with heterogeneous enhancement, blurred anatomy, and substantial noise preventing confident diagnosis; and 1 = very poor, with markedly heterogeneous enhancement, indistinct anatomy, severe noise, and non-diagnostic image quality. Scores of 3 or higher were considered diagnostically acceptable.

### 2.6 Comprehensive assessment

The two radiologists integrated the objective measurements and subjective image-quality scores across the evaluated venous levels to determine the optimal VMI energy level and ASIR-V strength for IVC-CTA.

### 2.7 Diagnostic confidence assessment

The same two radiologists independently assessed diagnostic confidence for all patients while blinded to the scanning protocol. The evaluation included: (1) presence of IVC thrombosis and clarity of thrombus margins; (2) assessability of luminal stenosis severity (mild, <50%; moderate, 50%–75%; severe, >75%); (3) position, morphology, and displacement of an IVC filter; and (4) presence of a stent, when applicable, and its patency. Diagnostic confidence was rated on a 5-point scale: 5 = lesion depicted extremely clearly, with very high diagnostic confidence; 4 = clearly depicted, with high confidence; 3 = adequately depicted for basic diagnosis; 2 = poorly depicted, with insufficient confidence; and 1 = not depicted or not assessable. Scores of 3 or higher were considered clinically acceptable. It should be noted that the diagnostic confidence score was a subjective measure of image interpretability and readers' confidence in image interpretation, rather than a measure of diagnostic accuracy or diagnostic performance.

### 2.8 Statistical analysis

Statistical analyses were performed using SPSS version 27.0 (IBM Corp., Armonk, NY, USA). A two-sided alpha level of 0.05 was used, and *P* < 0.05 was considered statistically significant. Normality of continuous variables was assessed using the Shapiro–Wilk test, and homogeneity of variance using Levene's test. Normally distributed continuous variables are presented as mean ± standard deviation, whereas non-normally distributed variables are presented as median (first quartile, third quartile). Categorical variables are reported as counts and percentages. Between-group differences in age, height, weight, BMI, radiation-dose metrics, objective image-quality parameters, and diagnostic-confidence scores were assessed using the independent-samples *t*-test for normally distributed variables or the Mann–Whitney *U*-test for non-normally distributed variables. Sex and IVC disease categories were compared using the chi-square test. Within Group B, repeated measurements across VMI levels (40–70 keV) and ASIR-V strengths (FBP to 100%) were analyzed using repeated-measures analysis of variance for normally distributed variables or the Friedman test for non-normally distributed variables; Bonferroni correction was applied to *post hoc* pairwise comparisons. Inter-observer agreement for subjective scores was assessed using the weighted kappa coefficient: 0.81 ≤ kappa < 1.00 indicated strong agreement; 0.61 ≤ kappa < 0.81, substantial agreement; 0.41 ≤ kappa < 0.61, moderate agreement; 0.21 ≤ kappa < 0.41, fair agreement; and kappa < 0.21, poor agreement. In addition, the subgroup analyses of diagnostic confidence across different lesion subgroups were exploratory and not powered to detect statistically significant differences; these results should therefore be interpreted as hypothesis-generating only.

## 3 Results

### 3.1 Demographic and clinical characteristics

No significant differences were observed between Groups A and B in age, weight, height, BMI, sex, or IVC disease category (all *P* > 0.05; Table 1). Across the entire cohort, 27 patients had thrombosis, 33 had stenosis, 39 had undergone IVC filter placement, and 21 had no IVC abnormality.

Radiation-dose metrics were significantly lower in Group B than in Group A. Median DLP was 671.75 mGy·cm (IQR, 650.59–687.10) in Group A vs. 587.64 mGy·cm (IQR, 565.58–607.33) in Group B (*P* < 0.001), and median ED was 10.07 mSv (IQR, 9.75–10.30) vs. 8.81 mSv (IQR, 8.48–9.11), respectively (*P* < 0.001). Contrast medium volume and iodine intake were also significantly lower in Group B: median contrast medium volume was 93.75 mL (IQR, 89.25–98.06) in Group A vs. 75.00 mL (IQR, 70.20–82.05) in Group B (*P* < 0.001), and median iodine intake was 37.50 g (IQR, 35.70–39.22) vs. 24.00 g (IQR, 22.46–26.25), respectively (*P* < 0.001). Compared with Group A, Group B showed reductions of approximately 12.51% in ED, 20.0% in contrast medium volume, and 36.0% in iodine intake (all *P* < 0.001; Figure 1).

**Figure 1:**
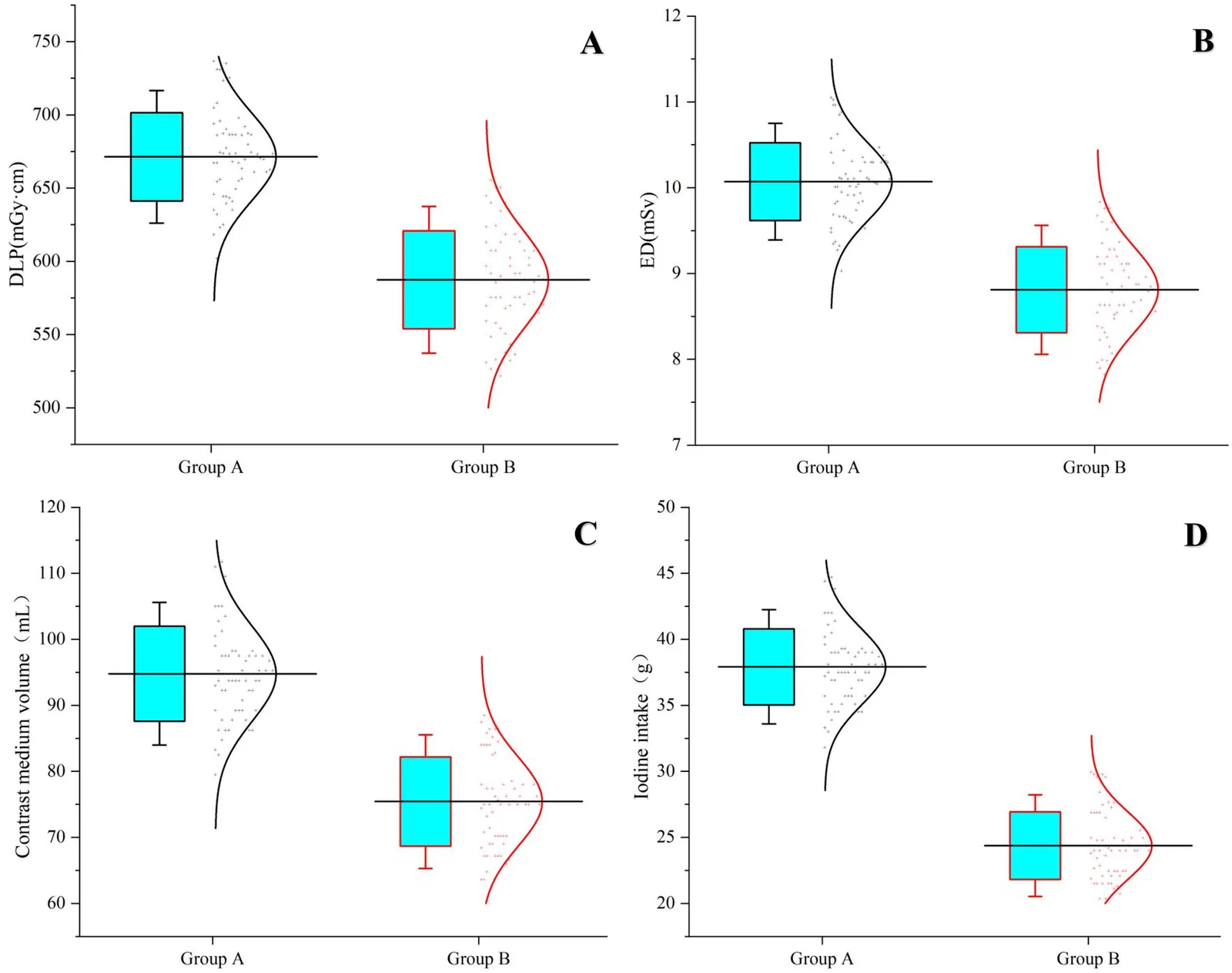
Comparison of dose and contrast-medium metrics between the conventional 120 kVp and DECT protocols. Compared with the 120 kVp protocol, DECT CTA showed significantly lower dose-length product **(A)**, effective radiation dose **(B)**, contrast medium volume **(C)**, and iodine intake **(D)** (all *P* < 0.001). Boxes represent the first and third quartiles, and whiskers extend to the 5th and 95th percentiles. The overlaid normal curves illustrate the distributional pattern. DECT = dual-energy computed tomography; CTA = computed tomography angiography; DLP = dose-length product; ED = effective dose.

### 3.2 Objective image quality across different VMI levels

In Group B, with ASIR-V fixed at 50%, CT attenuation, vascular SD, background noise, SNR, and CNR at all evaluated venous levels decreased progressively as VMI energy increased. The 40 keV dataset showed the highest CT attenuation, SNR, and CNR but also the greatest vascular SD and background noise, whereas the 70 keV dataset showed the lowest values. Pairwise comparisons with the other energy levels were statistically significant (all *P* < 0.05; Table 2 and Figure 2). Detailed results for all five measurement levels (hepatic IVC, suprarenal IVC, infrarenal IVC, common iliac vein, and femoral vein) are provided in Supplementary Table S1.

**Table 2:** CT attenuation, SD, background noise, SNR, and CNR in the hepatic IVC across VMI energy levels in group B.

Parameters	40 keV	45 keV	50 keV	55 keV	60 keV	65 keV	70 keV	*χ*^2^ value	*P* value
CT attenuation (HU)	433.75 (410.72, 453.37)	357.00 (338.40, 373.75)	295.65 (279.90, 307.47)	247.70 (235.32, 257.12)	210.25 (200.95, 216.82)	180.50 (173.85, 186.75)	156.75 (151.35, 162.77)	360.000	<0.001
SD (HU)	36.45 (34.17, 38.25)	30.75 (28.82, 32.17)	26.30 (24.50, 27.47)	22.65 (21.20, 23.67)	19.85 (18.70, 20.90)	17.75 (16.82, 18.57)	16.00 (15.12, 16.80)	360.000	<0.001
Background noise (HU)	32.25 (29.52, 33.47)	27.05 (24.75, 28.17)	22.85 (21.12, 23.87)	19.65 (18.20, 20.57)	17.30 (15.92, 18.10)	15.40 (14.25, 16.17)	13.85 (12.72, 14.65)	360.000	<0.001
SNR	11.82 (11.45, 12.24)	11.53 (11.22, 11.97)	11.19 (10.89, 11.64)	10.93 (10.56, 11.28)	10.58 (10.27, 10.92)	10.19 (9.87, 10.50)	9.80 (9.50, 10.15)	358.314	<0.001
CNR	10.20 (9.45, 11.29)	9.78 (9.00, 10.84)	9.31 (8.53, 10.23)	8.82 (8.00, 9.64)	8.16 (7.42, 8.98)	7.56 (6.83, 8.21)	6.99 (6.33, 7.71)	358.736	<0.001

Data are presented as median (first quartile, third quartile).

Detailed results for the hepatic IVC, suprarenal IVC, infrarenal IVC, common iliac vein, and femoral vein are provided in Supplementary Table S1.

CT, computed tomography; HU, Hounsfield unit; SD, standard deviation; SNR, signal-to-noise ratio; CNR, contrast-to-noise ratio; IVC, inferior vena cava; VMI, virtual monoenergetic imaging.

**Figure 2:**
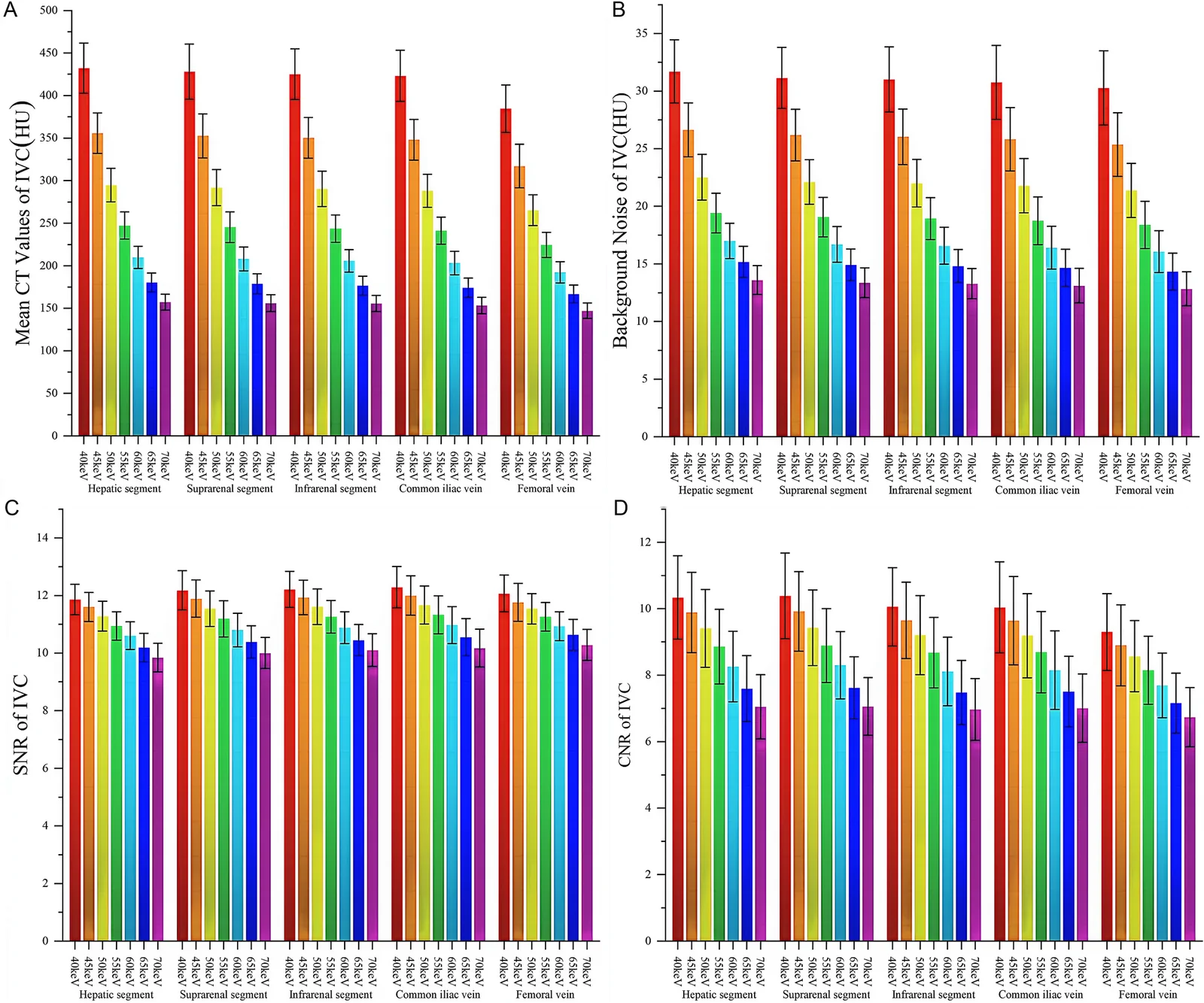
Objective image-quality parameters across VMI energy levels. Panels **A-D** show grouped bar charts comparing mean CT attenuation, background noise, SNR, and CNR across the evaluated venous levels at seven VMI energies (40-70 keV). CT attenuation, background noise, SNR, and CNR all decreased as VMI energy increased. Data are presented as means (bars) with errors of the mean (whiskers). VMI = virtual monoenergetic imaging; CT = computed tomography; SNR = signal-to-noise ratio; CNR = contrast-to-noise ratio.

### 3.3 Subjective image quality across different VMI levels

Inter-observer agreement for subjective IVC image-quality scores was substantial (weighted kappa values: 0.733, 0.833, 0.795, 0.923, 0.880, 0.938, and 0.860, respectively, all *P* > 0.05). For radiologist one, scores for the seven VMI datasets from 40 to 70 keV were 3.08 ± 0.27, 3.41 ± 0.49, 3.83 ± 0.37, 4.30 ± 0.46, 4.81 ± 0.39, 4.16 ± 0.37, and 3.65 ± 0.48, respectively (*χ*^2^ = 285.314, *P* < 0.001). For radiologist two, the corresponding scores were 3.05 ± 0.21, 3.50 ± 0.50, 3.88 ± 0.32, 4.33 ± 0.47, 4.85 ± 0.36, 4.15 ± 0.36, and 3.58 ± 0.49, respectively (*χ*^2^ = 287.161, *P* < 0.001). The 60 keV dataset had the highest subjective score and differed significantly from each of the other datasets (all *P* < 0.05). Integrating objective and subjective assessments, both radiologists considered 60 keV the optimal VMI energy level for IVC-CTA within the 40–70 keV range evaluated in this study (Figure 3).

**Figure 3:**
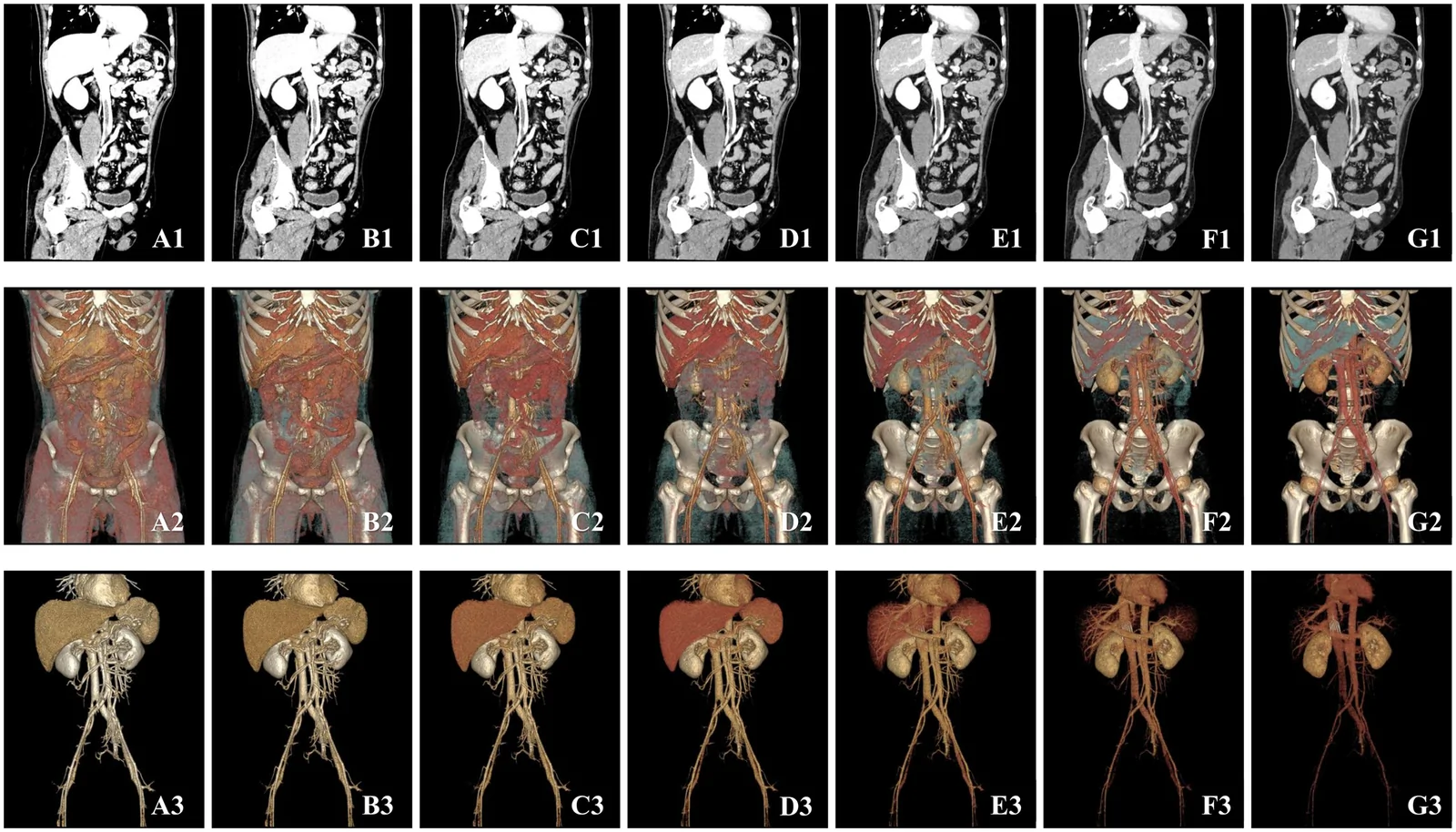
IVC images reconstructed at different VMI energy levels (40–70 keV in 5 keV increments) with 50% ASIR-V. **(A1–G1)** show coronal images reconstructed at 40, 45, 50, 55, 60, 65, and 70 keV, respectively. **(A2–G2)** and **(A3–G3)** show the corresponding bone-included and bone-removed VR images. As VMI energy increased, vascular contrast decreased and image noise was reduced; overall image quality was highest at 60 keV. IVC = inferior vena cava; VMI = virtual monoenergetic imaging; ASIR = adaptive statistical iterative reconstruction-V; VR = volume rendering.

### 3.4 Objective image quality across different ASIR-V levels

In Group [ABS]B, at a fixed VMI energy of 60 keV, CT attenuation at all evaluated venous levels did not change significantly with increasing ASIR-V strength (all *P* > 0.05). In contrast, vascular SD and background noise decreased progressively, whereas SNR and CNR increased. The FBP dataset differed significantly from the ASIR-V datasets in SD, background noise, SNR, and CNR (all *P* < 0.001). FBP yielded the highest SD and background noise, whereas 100% ASIR-V yielded the highest SNR and CNR (Table 3 and Figure 4). Detailed results for all five measurement levels are provided in Supplementary Table S2.

**Table 3:** CT attenuation, SD, background noise, SNR, and CNR in the hepatic IVC at 60 keV across reconstruction strengths in group B.

Parameters	FBP	ASIR-V 20%	ASIR-V 40%	ASIR-V 60%	ASIR-V 80%	ASIR-V 100%	χ^2^ value	*P* value
CT attenuation (HU)	210.25 (200.95, 216.82)	210.40 (200.92, 216.90)	210.40 (200.62, 216.85)	210.40 (200.90, 217.12)	209.75 (200.12, 217.10)	210.35 (200.47, 217.30)	5.244	0.387
SD (HU)	33.85 (32.40, 35.95)	28.35 (27.02, 29.57)	22.40 (21.20, 23.57)	16.80 (15.95, 17.75)	11.50 (10.90, 12.35)	7.40 (6.80, 8.47)	298.867	<0.001
Background noise (HU)	30.35 (28.62, 31.80)	25.15 (23.40, 26.57)	19.70 (18.07, 20.87)	14.80 (13.47, 15.40)	10.50 (9.62, 11.27)	7.90 (6.92, 8.80)	299.438	<0.001
SNR	6.16 (5.91, 6.42)	7.41 (7.18, 7.63)	9.35 (9.03, 9.61)	12.42 (12.13, 12.82)	18.15 (17.27, 18.73)	28.48 (25.51, 30.54)	298.867	<0.001
CNR	4.59 (4.19, 5.01)	5.56 (4.99, 6.10)	7.19 (6.45, 7.90)	9.71 (8.78, 10.46)	13.31 (12.06, 14.57)	17.75 (16.00, 19.99)	299.438	<0.001

Data are presented as median (first quartile, third quartile).

Detailed results for the hepatic IVC, suprarenal IVC, infrarenal IVC, common iliac vein, and femoral vein are provided in Supplementary Table S2.

CT, computed tomography; HU, Hounsfield unit; SD, standard deviation; SNR, signal-to-noise ratio; CNR, contrast-to-noise ratio; IVC, inferior vena cava; FBP, filtered back projection; ASIR-V, adaptive statistical iterative reconstruction-V.

**Figure 4:**
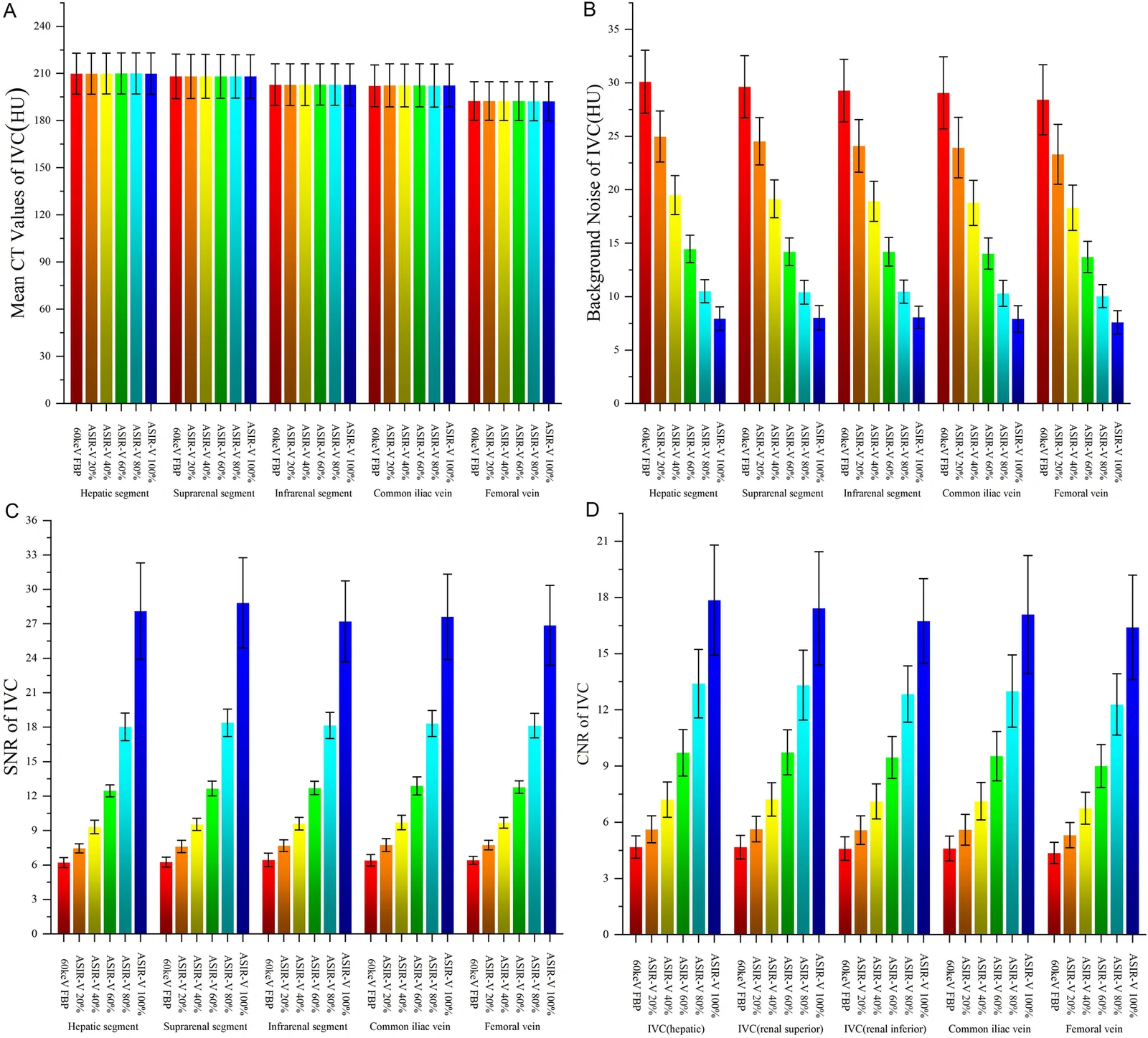
Objective image-quality parameters at 60 keV across different reconstruction strengths (FBP and ASIR-V 20%–100%). **(A–D)** show grouped bar charts comparing mean CT attenuation, background noise, SNR, and CNR across the evaluated venous levels. As ASIR-V strength increased, mean CT attenuation remained stable, background noise decreased, and SNR and CNR increased. Data are presented as means (bars) with errors of the mean (whiskers). FBP = filtered back projection; ASIR-V = adaptive statistical iterative reconstruction-V; CT = computed tomography; SNR = signal-to-noise ratio; CNR = contrast-to-noise ratio.

### 3.5 Subjective image quality across different ASIR-V levels

Inter-observer agreement for subjective IVC image-quality scores was substantial (weighted kappa values: 0.860, 0.757, 0.883, 0.793, 0.833, and 0.828, respectively, all *P* > 0.05). For radiologist one, scores for the six 60 keV datasets reconstructed with FBP and ASIR-V at 20%, 40%, 60%, 80%, and 100% were 3.35 ± 0.48, 3.76 ± 0.42, 4.28 ± 0.45, 4.83 ± 0.37, 4.50 ± 0.50, and 3.98 ± 0.46, respectively (*χ*^2^ = 227.790, *P* < 0.001). For radiologist two, the corresponding scores were 3.41 ± 0.49, 3.66 ± 0.47, 4.33 ± 0.47, 4.76 ± 0.42, 4.41 ± 0.49, and 4.05 ± 0.46, respectively (*χ*^2^ = 225.058, *P* < 0.001). The 60% ASIR-V dataset had the highest subjective score and differed significantly from all other reconstruction strengths (all *P* < 0.05). Based on the combined objective and subjective assessments, both radiologists considered 60% ASIR-V the optimal reconstruction strength for IVC-CTA among the FBP-to-100% ASIR-V range evaluated in this study (Figure 5).

**Figure 5:**
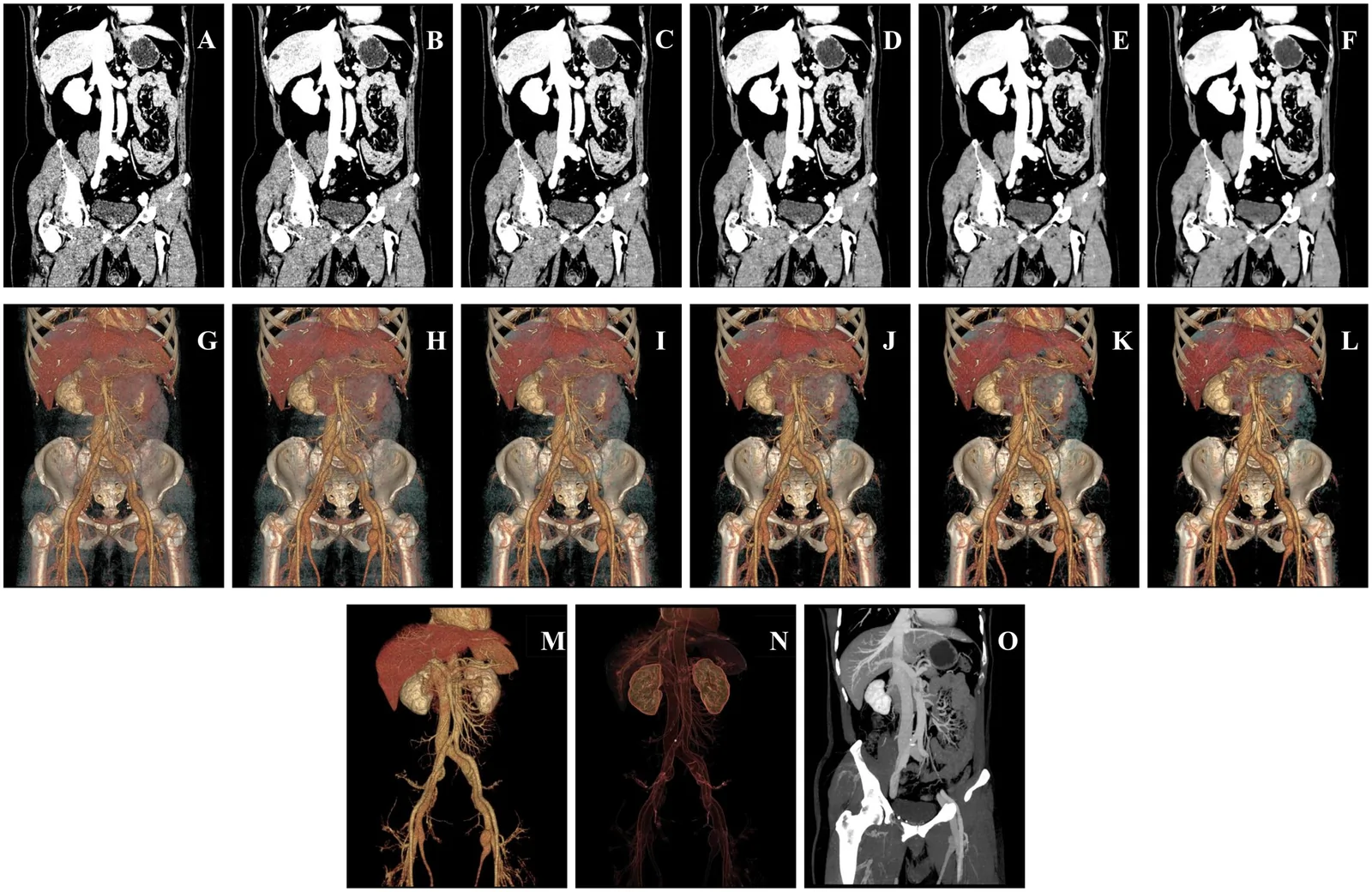
IVC images reconstructed at 60 keV using different ASIR-V strengths (FBP to 100% in 20% increments). **(A–F)** show coronal images reconstructed with FBP and ASIR-V at 20%, 40%, 60%, 80%, and 100%, respectively; **(G–L)** show the corresponding bone-included VR images; and **(M–O)** show bone-removed VR and MIP images from the 60 keV plus 60% ASIR-V dataset. Images are arranged from lower to higher reconstruction strength. Noise progressively decreased and vascular conspicuity improved from FBP to 60% ASIR-V, whereas strengths above 60% produced an overly smooth, waxy appearance. The 60% ASIR-V setting provided the most favorable balance between noise suppression and preservation of natural image texture. IVC = inferior vena cava; ASIR-V = adaptive statistical iterative reconstruction-V; FBP = filtered back projection; VR = volume rendering; MIP = maximum intensity projection.

### 3.6 Comprehensive evaluation

Within the stepwise optimization process, 60 keV VMI combined with 60% ASIR-V provided the most favorable balance among objective image quality, subjective scores, and image texture. This parameter combination was therefore defined as Group B1.

### 3.7 Comparison of image quality between groups A and B1

IVC CT attenuation, vascular SD, and background noise were significantly higher with the optimized DECT protocol (Group B1) than with the conventional protocol (Group A). However, SNR and CNR did not differ significantly between the two groups (*P* > 0.05). Mean subjective image-quality scores were 4.75 ± 0.43 in Group A and 4.80 ± 0.40 in Group B1. Inter-observer agreement was strong, with weighted kappa values of 0.857 (95% CI, 0.701–1.000) and 0.839 (95% CI, 0.664–1.000), respectively (both *P* < 0.001). Results are shown in Table 4 and Figure 6. Detailed data for all five measurement levels are provided in Supplementary Table S3.

**Table 4:** CT attenuation, SD, background noise, SNR, and CNR in the hepatic IVC in groups A and B1.

Parameters	Group A (120 kVp +50% ASIR-V)	Group B1 (60 keV +60% ASIR-V)	*Z* value	*P* value
CT attenuation (HU)	190.40 (182.80, 197.20)	210.40 (200.92, 216.90)	−7.464	<0.001
SD (HU)	15.40 (14.70, 16.00)	16.80 (15.95, 17.75)	−6.685	<0.001
Background noise (HU)	12.00 (11.50, 12.47)	14.80 (13.47, 15.40)	−8.473	<0.001
SNR	12.55 (11.84, 13.17)	12.42 (12.13, 12.82)	−0.546	0.585
CNR	9.71 (9.33, 10.37)	9.71 (8.78, 10.46)	−0.714	0.475

Data are presented as median (first quartile, third quartile).

Detailed results for the hepatic IVC, suprarenal IVC, infrarenal IVC, common iliac vein, and femoral vein are provided in Supplementary Table S3.

CT, computed tomography; HU, Hounsfield unit; SD, standard deviation; SNR, signal-to-noise ratio; CNR, contrast-to-noise ratio; IVC, inferior vena cava; ASIR-V, adaptive statistical iterative reconstruction-V.

**Figure 6:**
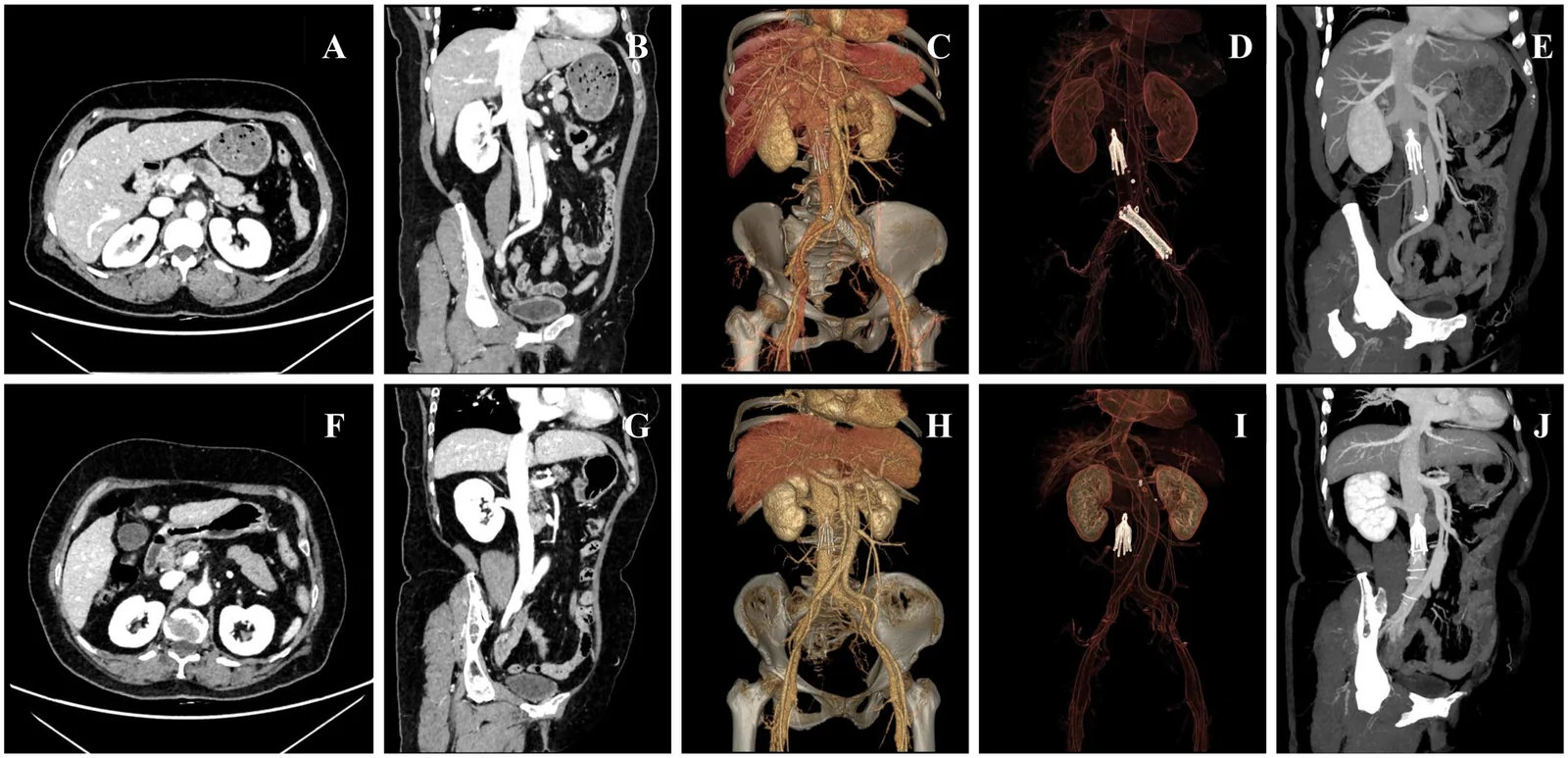
Comparison of IVC image quality between the conventional protocol and the optimized DECT protocol. **(A–E)** show the conventional protocol (120 kVp +400 mgI/mL +1.5 mL/kg +50% ASIR-V), and **(F–J)** show the optimized DECT protocol (60 keV +320 mgI/mL +1.2 mL/kg +60% ASIR-V), including axial, coronal, VR, and MIP images. Both protocols clearly depict vascular anatomy with sharp vessel margins; the subjective image-quality score was 5 for both. IVC = inferior vena cava; DECT = dual-energy computed tomography; ASIR-V = adaptive statistical iterative reconstruction-V; VR = volume rendering; MIP = maximum intensity projection.

### 3.8 Comparison of diagnostic confidence for specific findings between groups A and B1

The distribution of IVC findings did not differ significantly between the two groups (Table 1). For thrombosis, luminal stenosis, post-filter placement, and normal IVC findings, inter-observer agreement for diagnostic-confidence scores was substantial in both Groups A and B1 (weighted kappa ≥ 0.61). No thrombus was missed and no filter was misclassified in either group. Only Group A included patients with IVC stents (*n* = 2, both categorized within the filter-placement group), whereas Group B had no stent cases; therefore, stent-related diagnostic confidence was not compared. As shown in Table 5, diagnostic-confidence scores did not differ significantly between Groups A and B1 for any evaluated finding (all *P* > 0.05), suggesting that the optimized DECT protocol may provide lesion conspicuity and diagnostic confidence similar to those of the conventional protocol for the common IVC findings represented in this cohort.

**Table 5:** Diagnostic-confidence scores for IVC findings in groups A and B1.

Lesion type	Group A (n)	Group B1 (n)	Group A score	Group B1 score	*Z* value	*P* value
Thrombus	12	15	4.00 (4.00, 4.75)	4.00 (3.00, 4.00)	−0.570	0.569
Stenosis	18	15	4.00 (3.00, 5.00)	4.00 (3.00, 5.00)	−0.422	0.673
Filter placement	21	18	4.00 (3.50, 5.00)	4.00 (4.00, 5.00)	−0.742	0.458
Normal IVC	9	12	4.00 (4.00, 5.00)	5.00 (4.00, 5.00)	−0.615	0.538

Data are presented as median (first quartile, third quartile).

IVC, inferior vena cava.

Subgroup analyses were exploratory and not powered to detect statistically significant differences.

## 4 Discussion

Digital subtraction angiography (DSA), color Doppler flow imaging (CDFI), magnetic resonance imaging (MRI), and CTA are all used to evaluate IVC abnormalities. Although DSA is regarded as the reference standard for IVC disorders, its invasiveness makes it unsuitable as a first-line screening examination. CDFI is convenient and noninvasive and can provide hemodynamic information; however, it is highly operator dependent and susceptible to interference from bowel gas and adipose tissue. MRI offers excellent soft-tissue contrast but is relatively time-consuming and costly, and image quality may be degraded by respiratory and motion artifacts (19). Consequently, each of these techniques has limitations for comprehensive assessment of IVC disease.

By comparison, CTA is rapid and noninvasive and offers high spatial resolution, making it one of the most widely used and effective techniques for IVC disease. Conventional IVC CT venography can be performed using either a direct or an indirect approach. In direct CT venography (20), a diluted mixture of nonionic contrast medium and saline (typically 1:4–1:6) is injected simultaneously through dorsal veins of both feet. Because contrast medium enters the lower-extremity venous system directly and ascends toward the IVC, intravascular iodine concentration is high, three-dimensional reconstruction is effective, and the total amount of contrast medium can be reduced. However, the procedure is cumbersome. Inadequate systemic dilution may produce excessively high intraluminal iodine concentrations and beam-hardening artifacts, whereas incomplete mixing of contrast material and blood may cause laminar-flow artifacts. In patients with known or suspected IVC thrombosis, high-pressure injection may also pose a theoretical risk of thrombus dislodgement. Indirect CT venography (20, 21), in contrast, uses contrast injection through an upper-extremity vein and images the IVC after systemic recirculation. This approach is simpler and more practical and is therefore commonly used. However, substantial dilution during circulation reduces venous iodine concentration and tissue contrast, which can be particularly problematic for long-segment, non-occlusive IVC thrombi. Thus, image quality can be variable with both approaches. Previous strategies to improve venous enhancement have included increasing iodine concentration, increasing injected volume, or lowering tube voltage (7, 8, 22, 23). Although these strategies can improve image quality, they may increase iodine exposure, CI-AKI risk, and other adverse effects. Moreover, conventional CT performed at a single tube voltage (typically 70–140 kVp) yields a fixed polychromatic dataset with relatively limited spectral information. DECT offers an opportunity to overcome some of these limitations.

The DECT system used in this study employs rapid single-source kVp switching between 80 and 140 kVp, enabling near-simultaneous acquisition of low- and high-energy data from the same source. By exploiting differences in material attenuation across X-ray energies, a single acquisition can generate both conventional polychromatic images and 101 continuous VMI datasets from 40 to 140 keV. At lower energies, the photoelectric effect near the iodine K-edge (33.2 keV) markedly increases iodine attenuation, thereby enhancing vessel-to-background contrast. However, lower-energy photons have reduced penetrating power, resulting in fewer detected photons and greater quantum noise. Therefore, the optimal VMI energy for a given vascular task requires a balance between vascular contrast and image noise (24). Previous studies have generally identified the 40–60 keV range as favorable for CTA applications (14, 15, 18, 25–27). Lai et al. (26) reported that 60 keV VMI provided optimal image quality for superior mesenteric artery assessment while reducing iodine intake. Mao et al. (27) found that 60 keV VMI was optimal for CT enterography in inflammatory bowel disease and improved diagnostic confidence for active lesions. Chung et al. (28) similarly reported superior image quality at 60 keV in dual-energy CT arthrography of the wrist. By contrast, relatively few studies have addressed monoenergetic optimization for the IVC, with most venous work focusing on lower-extremity deep veins or the portal venous system (29, 30), whereas arterial applications are more widely reported. In the present study, 40 keV VMI produced the highest CT attenuation, SNR, and CNR, but also the greatest vascular SD and background noise and the lowest subjective image-quality score. This likely reflects the proximity of 40 keV to the iodine K-edge, which markedly enhances iodine signal but also amplifies quantum noise, resulting in coarse image texture. Thus, SNR or CNR alone should not be used to define the optimal image; objective metrics, subjective image quality, and image texture should be considered together. Within the 40–70 keV range evaluated in this study, 60 keV VMI provided the best overall balance between signal and noise across the evaluated venous levels, maintained adequate vascular contrast for diagnosis, and achieved the highest subjective score. These findings are broadly consistent with previous reports (28–30).

ASIR-V is an advanced iterative reconstruction algorithm that uses iterative forward- and back-projection processes to refine image data and allows the operator to select reconstruction strengths from 0% to 100%. Increasing the ASIR-V percentage generally increases noise suppression. Compared with conventional FBP and earlier-generation adaptive statistical iterative reconstruction, ASIR-V has shown advantages in reducing image noise and improving image quality (12, 13, 31, 32). After identifying 60 keV as the preferred VMI level, we further compared six reconstruction settings ranging from FBP to 100% ASIR-V. As ASIR-V strength increased, vascular SD and background noise progressively decreased, whereas SNR and CNR increased. Subjective image-quality scores increased from FBP through 60% ASIR-V but decreased at strengths above 60%. Although very high ASIR-V strengths, such as 100%, substantially suppressed noise and yielded the highest SNR and CNR, they also produced excessive smoothing and loss of natural noise texture, resulting in an artificial plastic-like or waxy appearance and lower reader scores. Thus, the numerically highest objective metrics did not correspond to the best clinical appearance. Within the reconstruction range evaluated in this study, 60% ASIR-V provided the most favorable balance between noise reduction and preservation of natural image texture. Pontone et al. (33) reported a similar balance with 60% ASIR-V in coronary CTA. Zhao et al. (34) suggested that, in pediatric abdominal CT, combinations incorporating 60% post-reconstruction ASIR-V were optimal for both unenhanced and contrast-enhanced imaging. Li et al. (35) also showed that automatic kVp selection combined with 60% ASIR-V enabled individualized low-dose chest CT while maintaining image quality and diagnostic accuracy. These findings support the practical utility of an intermediate ASIR-V strength in clinical imaging.

Image quality and radiation dose remain central concerns when DECT is used for vascular imaging. A distinctive feature of the present study was the systematic integration of a reduced-concentration/reduced-volume contrast strategy (320 mgI/mL at 1.2 mL/kg) with VMI energy optimization and ASIR-V strength optimization, followed by direct comparison with a conventional protocol using 400 mgI/mL at 1.5 mL/kg. Although the optimized 60 keV VMI plus 60% ASIR-V protocol (Group B1) produced slightly higher vascular SD and background noise than Group A, SNR, CNR, and subjective image-quality scores were similar between groups. This may reflect complementary effects of the two techniques: 60 keV VMI increases iodine conspicuity and vessel contrast, whereas 60% ASIR-V suppresses noise while preserving diagnostically useful image texture, thereby maintaining SNR and CNR at clinically acceptable levels. We also evaluated diagnostic confidence for specific findings. Scores for thrombosis, luminal stenosis, and filter placement were not significantly different between the optimized DECT and conventional 120 kVp protocols, suggesting that the reduced iodine and radiation exposure did not compromise reader confidence in this cohort. Compared with Group A, the optimized DECT protocol reduced contrast medium volume by approximately 20.0%, iodine intake by 36.0%, and ED by 12.51%. These results suggest that the optimized protocol can reduce iodine load while also lowering radiation exposure relative to the conventional protocol.

Several limitations should be acknowledged. First, this was a non-randomized, single-center feasibility study with a relatively small sample size; optimization and evaluation were performed in the same cohort, and no reference standard or equivalence design was used. Therefore, the absence of statistically significant differences should not be interpreted as equivalence, diagnostic confidence should not be equated with diagnostic accuracy, and the identified protocol combining 60 keV VMI with 60% ASIR-V should be considered hypothesis-generating. Further validation, ideally through multicenter randomized controlled trials with larger cohorts and an appropriate reference standard, is warranted. Second, all data were acquired and reconstructed on a single vendor's DECT platform, and generalizability to other systems remains to be established. Third, only ASIR-V was evaluated; other reconstruction methods, including deep learning image reconstruction, warrant further investigation. Fourth, because the optimal keV level was first selected at 50% ASIR-V and the optimal ASIR-V strength was then determined at that energy, potential interactions between keV and reconstruction strength were not fully assessed, and a full-factorial analysis was not performed. Finally, renal function parameters such as serum creatinine and estimated glomerular filtration rate were not measured before or after contrast administration. Therefore, the observed reduction in iodine load cannot be directly linked to a lower incidence of CI-AKI and should be interpreted only as a potential renal-sparing effect. Future studies with dedicated renal safety endpoints are needed to confirm this potential benefit.

## 5 Conclusion

In conclusion, under the conditions of this study, a DECT protocol combining 60 keV VMI with 60% ASIR-V provided a favorable balance of image quality for IVC-CTA. The optimized protocol achieved SNR, CNR, subjective image quality, and diagnostic confidence similar to those of a conventional 120 kVp protocol while reducing contrast medium volume, iodine intake, and effective radiation dose. This approach may offer a practical option for routine IVC evaluation, particularly in patients requiring repeated imaging, filter or thrombosis follow-up, or those requiring careful contrast medium management.

## Data Availability

The original contributions presented in the study are included in the article/Supplementary Material, further inquiries can be directed to the corresponding author/s.
