# Supplementary material for: Optimized dual-energy CT protocol for inferior vena cava CT angiography using 60 keV virtual monoenergetic imaging and 60% ASIR-V
Source: Front Cardiovasc Med. 2026 Oct 29;13:1969018. doi: 10.3389/fcvm.2026.1969018 (PMC13754684; doi:10.3389/fcvm.2026.1969018)
Supplement: Supplementary file 1 [file Supplementaryfile1.docx]

**Supplementary Figure 1.** Flow diagram of the study. IVC = inferior vena cava; CTA = computed tomography angiography; DECT = dual-energy computed tomography; VMI = virtual monoenergetic images; ASIR-V = adaptive statistical iterative reconstruction-V; VR = volume rendering, MPR = multiplanar reconstruction, MIP = maximum intensity projection.

**C**

**B**

**A**

**E**

**D**

**Supplementary Figure 2.** Placement of ROIs for objective measurements of IVC image quality. Panels A-E show ROIs placement in the hepatic IVC, suprarenal IVC, infrarenal IVC, common iliac vein, and femoral vein, respectively, together with the corresponding ROIs in muscle at the same axial level. ROIs = region of interests; IVC = inferior vena cava.
