# Supplementary material for: Optimized dual-energy CT protocol for inferior vena cava CT angiography using 60 keV virtual monoenergetic imaging and 60% ASIR-V
Source: Front Cardiovasc Med. 2026 Oct 29;13:1969018. doi: 10.3389/fcvm.2026.1969018 (PMC13754684; doi:10.3389/fcvm.2026.1969018)
Supplement: Supplementary file 2 [file Table1.docx]

| **Supplementary Table S1. CT attenuation, SD, background noise, SNR, and CNR at different IVC levels across VMI energies in Group B** | | | | | | | | | |
| --- | --- | --- | --- | --- | --- | --- | --- | --- | --- |
| **Parameters** | **40 keV** | **45 keV** | **50 keV** | **55 keV** | **60 keV** | **65 keV** | **70 keV** | **χ^2^ value** | **P value** |
| Hepatic IVC | | | | | | | | | |
| CT attenuation (HU) | 433.75 (410.72, 453.37) | 357.00 (338.40, 373.75) | 295.65 (279.90, 307.47) | 247.70 (235.32, 257.12) | 210.25 (200.95, 216.82) | 180.50 (173.85, 186.75) | 156.75 (151.35, 162.77) | 360.000 | < 0.001 |
| SD (HU) | 36.45 (34.17, 38.25) | 30.75 (28.82, 32.17) | 26.30 (24.50, 27.47) | 22.65 (21.20, 23.67) | 19.85 (18.70, 20.90) | 17.75 (16.82, 18.57) | 16.00 (15.12, 16.80) | 360.000 | < 0.001 |
| Background noise (HU) | 32.25 (29.52, 33.47) | 27.05 (24.75, 28.17) | 22.85 (21.12, 23.87) | 19.65 (18.20, 20.57) | 17.30 (15.92, 18.10) | 15.40 (14.25, 16.17) | 13.85 (12.72, 14.65) | 360.000 | < 0.001 |
| SNR | 11.82 (11.45, 12.24) | 11.53 (11.22, 11.97) | 11.19 (10.89, 11.64) | 10.93 (10.56, 11.28) | 10.58 (10.27, 10.92) | 10.19 (9.87, 10.50) | 9.80 (9.50, 10.15) | 358.314 | < 0.001 |
| CNR | 10.20 (9.45, 11.29) | 9.78 (9.00, 10.84) | 9.31 (8.53, 10.23) | 8.82 (8.00, 9.64) | 8.16 (7.42, 8.98) | 7.56 (6.83, 8.21) | 6.99 (6.33, 7.71) | 358.736 | < 0.001 |
| Suprarenal IVC | | | | | | | | | |
| CT attenuation (HU) | 430.60 (405.50, 449.75) | 354.35 (335.07, 368.07) | 293.25 (277.57, 303.00) | 246.10 (232.65, 254.00) | 209.00 (199.22, 215.77) | 178.75 (172.35, 185.87) | 155.75 (150.65, 161.97) | 360.000 | < 0.001 |
| SD (HU) | 35.40 (33.00, 37.07) | 29.95 (27.92, 31.20) | 25.50 (23.82, 26.70) | 22.15 (20.70, 23.10) | 19.40 (18.20, 20.37) | 17.45 (16.30, 18.20) | 15.75 (14.70, 16.57) | 360.000 | < 0.001 |
| Background noise (HU) | 30.60 (29.60, 32.97) | 25.75 (24.90, 27.57) | 21.95 (21.00, 23.35) | 19.00 (18.02, 20.22) | 16.70 (15.70, 17.67) | 14.95 (14.00, 15.92) | 13.35 (12.50, 14.25) | 360.000 | < 0.001 |
| SNR | 12.10 (11.72, 12.53) | 11.80 (11.47, 12.22) | 11.42 (11.14, 11.90) | 11.13 (10.80, 11.39) | 10.74 (10.44, 11.06) | 10.29 (10.03, 10.69) | 9.95 (9.67, 10.26) | 357.471 | < 0.001 |
| CNR | 10.63 (9.37, 11.18) | 10.14 (8.96, 10.68) | 9.57 (8.55, 10.15) | 8.97 (8.10, 9.56) | 8.34 (7.60, 9.01) | 7.70 (7.03, 8.25) | 7.15 (6.59, 7.56) | 358.314 | < 0.001 |
| Infrarenal IVC | | | | | | | | | |
| CT attenuation (HU) | 417.20 (395.47, 433.15) | 343.90 (326.95, 357.62) | 284.95 (271.65, 296.87) | 239.20 (229.10, 248.55) | 203.55 (195.27, 211.30) | 174.95 (168.90, 182.27) | 153.25 (147.92, 159.67) | 360.000 | < 0.001 |
| SD (HU) | 33.50 (32.45, 35.37) | 28.30 (27.45, 29.80) | 24.20 (23.42, 25.50) | 21.00 (20.20, 22.07) | 18.45 (17.82, 19.37) | 16.70 (16.00, 17.40) | 15.10 (14.40, 15.67) | 360.000 | < 0.001 |
| Background noise (HU) | 31.25 (29.05, 32.57) | 26.30 (24.42, 27.27) | 22.30 (20.55, 23.10) | 19.05 (17.70, 19.87) | 16.75 (15.40, 17.37) | 15.00 (13.72, 15.57) | 13.40 (12.32, 14.00) | 360.000 | < 0.001 |
| SNR | 12.16 (11.86, 12.56) | 11.85 (11.62, 12.22) | 11.51 (11.25, 11.93) | 11.19 (10.92, 11.61) | 10.80 (10.51, 11.15) | 10.32 (10.17, 10.69) | 10.02 (9.78, 10.36) | 358.300 | < 0.001 |
| CNR | 9.98 (9.00, 11.13) | 9.53 (8.64, 10.72) | 9.02 (8.23, 10.17) | 8.52 (7.80, 9.54) | 7.86 (7.27, 8.88) | 7.20 (6.82, 8.18) | 6.78 (6.37, 7.60) | 358.736 | < 0.001 |
| Common iliac vein | | | | | | | | | |
| CT attenuation (HU) | 413.95 (388.45, 437.57) | 341.60 (320.80, 360.27) | 283.40 (265.87, 297.15) | 238.10 (224.45, 248.62) | 202.60 (191.15, 211.65) | 175.00 (164.55, 182.70) | 153.30 (144.35, 160.07) | 360.000 | < 0.001 |
| SD (HU) | 33.30 (31.82, 35.15) | 28.15 (27.02, 29.75) | 24.10 (23.00, 25.37) | 20.90 (19.90, 21.97) | 18.30 (17.60, 19.37) | 16.55 (15.70, 17.30) | 14.90 (14.30, 15.77) | 360.000 | < 0.001 |
| Background noise (HU) | 30.50 (28.80, 33.15) | 25.60 (24.12, 27.90) | 21.60 (20.32, 23.57) | 18.60 (17.35, 20.30) | 16.20 (15.07, 17.87) | 14.50 (13.52, 15.90) | 13.00 (12.05, 14.30) | 360.000 | < 0.001 |
| SNR | 12.20 (11.76, 12.66) | 11.91 (11.47, 12.38) | 11.60 (11.19, 11.98) | 11.23 (10.87, 11.64) | 10.85 (10.56, 11.26) | 10.43 (10.14, 10.86) | 10.07 (9.80, 10.50) | 357.486 | < 0.001 |
| CNR | 10.06 (9.32, 10.81) | 9.61 (8.98, 10.39) | 9.14 (8.51, 9.99) | 8.63 (8.00, 9.51) | 8.12 (7.39, 8.97) | 7.48 (6.85, 8.24) | 6.95 (6.33, 7.68) | 358.729 | < 0.001 |
| Femoral vein | | | | | | | | | |
| CT attenuation (HU) | 383.15 (365.35, 402.37) | 317.80 (300.62, 332.75) | 266.00 (250.80, 277.00) | 223.65 (212.87, 233.67) | 191.70 (182.50, 200.42) | 166.90 (159.60, 172.95) | 147.45 (140.45, 151.27) | 359.579 | < 0.001 |
| SD (HU) | 32.00 (30.40, 33.40) | 27.00 (25.72, 28.27) | 23.05 (22.00, 24.10) | 20.00 (19.02, 20.97) | 17.60 (16.65, 18.47) | 15.65 (14.82, 16.50) | 14.40 (13.60, 15.07) | 360.000 | < 0.001 |
| Background noise (HU) | 30.35 (28.07, 32.30) | 25.45 (23.45, 27.10) | 21.55 (19.70, 22.87) | 18.40 (16.85, 19.60) | 16.00 (14.62, 17.17) | 14.30 (13.12, 15.30) | 12.80 (11.67, 13.77) | 360.000 | < 0.001 |
| SNR | 12.02 (11.73, 12.49) | 11.80 (11.47, 12.21) | 11.51 (11.24, 11.95) | 11.25 (10.93, 11.60) | 10.99 (10.55, 11.24) | 10.69 (10.20, 10.95) | 10.32 (9.92, 10.61) | 350.879 | < 0.001 |
| CNR | 9.24 (8.50, 9.71) | 8.88 (8.09, 9.44) | 8.47 (7.82, 9.09) | 8.03 (7.46, 8.74) | 7.61 (6.97, 8.24) | 7.09 (6.50, 7.69) | 6.56 (6.06, 7.30) | 348.657 | < 0.001 |
| Data are presented as median (first quartile, third quartile).  CT = computed tomography; HU = Hounsfield unit; SD = standard deviation; SNR = signal-to-noise ratio; CNR = contrast-to-noise ratio; IVC = inferior vena cava; VMI = virtual monoenergetic imaging | | | | | | | | | |

| **Supplementary Table S2. CT attenuation, SD, background noise, SNR, and CNR at different IVC levels on 60-keV VMI across reconstruction strengths in Group B** | | | | | | | | |
| --- | --- | --- | --- | --- | --- | --- | --- | --- |
| **Parameters** | **FBP** | **ASIR-V 20%** | **ASIR-V 40%** | **ASIR-V 60%** | **ASIR-V 80%** | **ASIR-V 100%** | **χ^2^ value** | ***P* value** |
| Hepatic IVC | | | | | | | | |
| CT attenuation (HU) | 210.25 (200.95, 216.82) | 210.40 (200.92, 216.90) | 210.40 (200.62, 216.85) | 210.40 (200.92, 216.90) | 209.75  (200.12, 217.10) | 210.35 (200.47, 217.30) | 5.780 | 0.328 |
| SD (HU) | 33.85 (32.40, 35.95) | 28.35 (27.02, 29.57) | 22.40 (21.20, 23.57) | 16.80 (15.95, 17.75) | 11.50 (10.90, 12.35) | 7.40 (6.80, 8.47) | 298.867 | < 0.001 |
| Background noise (HU) | 30.35 (28.62, 31.80) | 25.15 (23.40, 26.57) | 19.70 (18.07, 20.87) | 14.80 (13.47, 15.40) | 10.50 (9.62, 11.27) | 7.90 (6.92, 8.80) | 299.438 | < 0.001 |
| SNR | 6.16 (5.91, 6.42) | 7.41 (7.18, 7.63) | 9.35 (9.03, 9.61) | 12.42 (12.13, 12.82) | 18.15 (17.27, 18.73) | 28.48 (25.51, 30.54) | 298.867 | < 0.001 |
| CNR | 4.59 (4.19, 5.01) | 5.56 (4.99, 6.10) | 7.19 (6.44, 7.90) | 9.71 (8.78, 10.46) | 13.31 (12.06, 14.57) | 17.75 (16.00, 19.99) | 299.438 | < 0.001 |
| Suprarenal IVC | | | | | | | | |
| CT attenuation (HU) | 209.20 (198.42, 216.27) | 209.20 (198.77, 216.05) | 208.95 (199.12, 215.92) | 208.95 (199.40, 215.70) | 209.40 (199.65, 216.72) | 209.10 (199.90, 216.67) | 1.103 | 0.954 |
| SD (HU) | 33.85 (31.62, 35.20) | 27.95 (25.72, 29.30) | 22.10 (20.65, 23.27) | 16.55 (15.60, 17.40) | 11.25 (10.60, 12.07) | 7.15 (6.40, 8.10) | 300.000 | < 0.001 |
| Background noise (HU) | 29.70 (28.02, 31.20) | 24.40 (23.22, 25.77) | 18.95 (18.12, 20.27) | 14.30 (13.22, 15.10) | 10.40 (9.62, 11.20) | 7.85 (7.10, 8.70) | 299.438 | < 0.001 |
| SNR | 6.22 (5.95, 6.46) | 7.54 (7.22, 7.87) | 9.50 (9.20, 9.77) | 12.60 (12.32, 12.94) | 18.47 (17.63, 19.33) | 28.81 (26.28, 32.09) | 300.000 | < 0.001 |
| CNR | 4.70 (4.27, 5.07) | 5.68 (5.11, 6.03) | 7.34 (6.53, 7.84) | 9.80 (8.95, 10.51) | 13.72 (11.70, 14.74) | 17.98 (14.95, 19.65) | 299.438 | < 0.001 |
| Infrarenal IVC | | | | | | | | |
| CT attenuation (HU) | 203.25 (194.85, 211.35) | 203.35 (195.05, 211.20) | 203.40 (195.32, 211.22) | 203.40 (195.22, 211.20) | 203.50 (195.27, 211.35) | 203.55 (195.37, 211.47) | 0.361 | 0.996 |
| SD (HU) | 31.80 (30.40, 33.57) | 26.20 (25.05, 27.87) | 20.95 (20.22, 22.05) | 15.85 (15.30, 16.60) | 11.10 (10.50, 11.70) | 7.55 (6.60, 8.37) | 299.438 | < 0.001 |
| Background noise (HU) | 29.45 (27.90, 30.87) | 24.00 (22.50, 25.67) | 18.95 (17.57, 19.97) | 14.20 (13.32, 15.00) | 10.50 (9.72, 11.10) | 7.90 (7.40, 8.60) | 300.000 | < 0.001 |
| SNR | 6.31 (6.03, 6.66) | 7.57 (7.34, 7.97) | 9.53 (9.19, 9.84) | 12.61 (12.35, 13.07) | 18.14 (17.32, 18.90) | 26.70 (24.35, 30.12) | 299.438 | < 0.001 |
| CNR | 4.62 (4.01, 5.12) | 5.59 (4.96, 6.20) | 7.04 (6.43, 7.76) | 9.33 (8.54, 10.44) | 12.88 (11.58, 14.03) | 17.13 (14.81, 18.29) | 300.000 | < 0.001 |
| Common iliac vein | | | | | | | | |
| CT attenuation (HU) | 202.90 (190.87, 212.60) | 202.70 (191.02, 212.22) | 202.60 (191.20, 211.80) | 202.45 (191.27, 211.37) | 202.45 (191.50, 211.07) | 202.30 (191.62, 210.80) | 4.073 | 0.539 |
| SD (HU) | 31.30 (29.90, 33.30) | 25.90 (24.77, 27.77) | 20.75 (19.82, 22.10) | 15.60 (15.02, 16.40) | 11.00 (10.50, 11.60) | 7.40 (6.70, 8.07) | 300.000 | < 0.001 |
| Background noise (HU) | 29.10 (26.82, 31.15) | 23.90 (22.05, 25.75) | 18.60 (17.32, 20.35) | 13.95 (12.90, 15.17) | 10.20 (9.40, 11.20) | 7.90 (7.00, 8.50) | 300.000 | < 0.001 |
| SNR | 6.32 (6.07, 6.66) | 7.66 (7.30, 8.01) | 9.55 (9.29, 10.00) | 12.69 (12.42, 13.11) | 18.34 (17.57, 19.03) | 27.28 (25.31, 30.31) | 300.000 | < 0.001 |
| CNR | 4.55 (4.18, 4.91) | 5.53 (5.11, 6.04) | 7.01 (6.55, 7.73) | 9.61 (8.69, 10.38) | 13.28 (11.73, 14.34) | 16.95 (14.79, 19.19) | 300.000 | < 0.001 |
| Femoral vein | | | | | | | | |
| CT attenuation (HU) | 191.95 (182.72, 200.25) | 191.85 (182.55, 200.17) | 191.60 (182.42, 200.45) | 191.65 (182.35, 200.40) | 191.50 (182.20, 200.20) | 191.40 (182.22, 200.00) | 1.406 | 0.924 |
| SD (HU) | 30.05 (28.35, 31.92) | 24.95 (23.62, 26.30) | 20.05 (19.00, 21.05) | 15.15 (14.32, 15.87) | 10.55 (10.00, 11.17) | 7.10 (6.40, 7.80) | 300.000 | < 0.001 |
| Background noise (HU) | 28.30 (26.22, 30.55) | 23.40 (21.22, 25.27) | 18.20 (16.82, 19.67) | 13.75 (12.62, 14.70) | 9.95 (9.40, 10.77) | 7.60 (6.72, 8.20) | 300.000 | < 0.001 |
| SNR | 6.40 (6.16, 6.68) | 7.71 (7.48, 8.03) | 9.64 (9.36, 9.99) | 12.78 (12.45, 13.08) | 18.35 (17.48, 18.89) | 27.38 (24.24, 29.22) | 300.000 | < 0.001 |
| CNR | 4.22 (3.92, 4.70) | 5.18 (4.78, 5.63) | 6.73 (6.13, 7.21) | 8.82 (8.22, 9.58) | 12.34 (11.11, 13.11) | 16.25 (13.94, 18.54) | 300.000 | < 0.001 |
| Data are presented as median (first quartile, third quartile).  CT = computed tomography; HU = Hounsfield unit; SD = standard deviation; SNR = signal-to-noise ratio; CNR = contrast-to-noise ratio; FBP = filtered back projection; IVC = inferior vena cava; ASIR-V = adaptive statistical iterative reconstruction-V; VMI = virtual monoenergetic imaging | | | | | | | | |

| **Supplementary Table S3. CT attenuation, SD, background noise, SNR, and CNR at different IVC levels in Groups A and B1** | | | | |
| --- | --- | --- | --- | --- |
| **Parameters** | **Group A (120 kVp + 50% ASIR-V)** | **Group B1 (60 keV + 60% ASIR-V)** | **Z value** | ***P* value** |
| Hepatic IVC | | | | |
| CT attenuation (HU) | 190.40 (182.80, 197.20) | 210.40 (200.92, 216.90) | -7.464 | < 0.001 |
| SD (HU) | 15.40 (14.70, 16.00) | 16.80 (15.95, 17.75) | -6.685 | < 0.001 |
| Background noise (HU) | 12.00 (11.50, 12.47) | 14.80 (13.47, 15.40) | -8.473 | < 0.001 |
| SNR | 12.55 (11.84, 13.17) | 12.42 (12.13, 12.82) | -0.546 | 0.585 |
| CNR | 9.71 (9.33, 10.37) | 9.71 (8.78, 10.46) | -0.714 | 0.475 |
| Suprarenal IVC | | | | |
| CT attenuation (HU) | 187.85 (182.97, 190.32) | 208.95 (199.40, 215.70) | -7.477 | < 0.001 |
| SD (HU) | 14.95 (14.10, 15.70) | 16.55 (15.60, 17.40) | -6.394 | < 0.001 |
| Background noise (HU) | 11.90 (11.60, 12.40) | 14.30 (13.22, 15.10) | -8.703 | < 0.001 |
| SNR | 12.61 (12.05, 13.34) | 12.60 (12.32, 12.94) | -0.262 | 0.793 |
| CNR | 9.56 (9.23, 10.36) | 9.80 (8.95, 10.51) | -0.425 | 0.671 |
| Infrarenal IVC | | | | |
| CT attenuation (HU) | 185.55 (182.50, 190.62) | 203.40 (195.22, 211.20) | -6.131 | < 0.001 |
| SD (HU) | 15.35 (14.12, 15.70) | 15.85 (15.30, 16.60) | -4.364 | < 0.001 |
| Background noise (HU) | 12.30 (11.80, 12.50) | 14.20 (13.32, 15.00) | -7.673 | < 0.001 |
| SNR | 12.66 (11.71, 13.50) | 12.61 (12.35, 13.07) | -0.504 | 0.614 |
| CNR | 9.48 (8.98, 10.18) | 9.33 (8.54, 10.44) | -0.845 | 0.398 |
| Common iliac vein | | | | |
| CT attenuation (HU) | 186.80 (183.30, 189.05) | 202.45 (191.27, 211.37) | -5.921 | < 0.001 |
| SD (HU) | 14.50 (14.00, 14.85) | 15.60 (15.02, 16.40) | -6.398 | < 0.001 |
| Background noise (HU) | 11.75 (11.30, 12.40) | 13.95 (12.90, 15.17) | -7.663 | < 0.001 |
| SNR | 13.05 (12.18, 13.62) | 12.69 (12.42, 13.11) | -1.115 | 0.265 |
| CNR | 9.75 (9.29, 10.36) | 9.61 (8.69, 10.38) | -1.239 | 0.215 |
| Femoral vein | | | | |
| CT attenuation (HU) | 182.55 (178.05, 186.85) | 191.65 (182.35, 200.40) | -4.659 | < 0.001 |
| SD (HU) | 14.20 (13.72, 14.50) | 15.15 (14.32, 15.87) | -5.320 | < 0.001 |
| Background noise (HU) | 12.05 (11.52, 12.50) | 13.75 (12.62, 14.70) | -6.864 | < 0.001 |
| SNR | 12.81 (12.47, 13.31) | 12.78 (12.45, 13.08) | -0.829 | 0.407 |
| CNR | 8.96 (8.63, 9.86) | 8.82 (8.22, 9.58) | -1.475 | 0.140 |
| Data are presented as median (first quartile, third quartile).  CT = computed tomography; HU = Hounsfield unit; SD = standard deviation; SNR = signal-to-noise ratio; CNR = contrast-to-noise ratio; IVC = inferior vena cava; ASIR-V = adaptive statistical iterative reconstruction-V; VMI = virtual monoenergetic imaging | | | | |
